# MiR-497-5p down-regulates CDCA4 to restrains lung squamous cell carcinoma progression

**DOI:** 10.1186/s13019-021-01698-2

**Published:** 2021-11-12

**Authors:** Jiangwei Hu, Xinqin Xiang, Wei Guan, Weihua Lou, Junming He, Jian Chen, Yin Fu, Guoliang Lou

**Affiliations:** Department of Cardiovascular Surgery, Yiwu Central Hospital, No.699 Jiangdong Dong Lu, Yiwu City, 322000 Zhejiang Province China

**Keywords:** miR-497-5p, CDCA4, LUSC, Proliferation, Migration, Invasion

## Abstract

**Background:**

So far, few have concerned miR-497-5p in lung squamous cell carcinoma (LUSC).

**Methods:**

MiR-497-5p expression in LUSC was measured by qRT-PCR. Its impacts on tumor-related cell behaviors were investigated by CCK8 assay, scratch healing assay, flow cytometry and Transwell invasion methods. In addition, interaction between miR-497-5p and CDCA4 in LUSC was also elucidated through rescue experiment, western blot, dual-luciferase, and bioinformatics analysis.

**Results:**

Low level of miR-497-5p was confirmed in LUSC tissue and cells. Overexpressed miR-497-5p markedly inhibited cancer progression. miR-497-5p restrained CDCA4 expression. Rescue assay showed that overexpressing miR-497-5p eliminated effect of overexpressed CDCA4.

**Conclusion:**

By targeting CDCA4, miR-497-5p restrained development of LUSC.

**Supplementary Information:**

The online version contains supplementary material available at 10.1186/s13019-021-01698-2.

## Background

There are approximately 1,600,000 new lung cancer cases annually [1]. According to statistics, lung cancer makes up 17.09% of cancer cases in China, with a mortality of 24.35%, making it the most common and fatal type of all cancers [2]. In recent years, despite the rapid development of therapies, patient’s survival is still poor [3]. Because the genetic and epigenetic changes of lung squamous cell carcinoma (LUSC) are very different [4], individualized treatment strategies based on early symptoms of LUSC patients are required. Currently, detection of biomarkers for LUSC patients has been conducted to enhance patient’s survival [5], but the molecular mechanism related to LUSC has not been studied in depth. Based on the above facts, understanding the molecular mechanism of LUSC can provide theoretical help for searching for new LUSC therapies.

Many microRNAs (miRNAs) participate in the development of LUSC [6]. Chang et al*. *[7] found that miR-448 can regulate cell proliferation and inhibit cell apoptosis by targeting DCLK1 in LUSC cells. Wang et al*. *[8] revealed that miR-372-3p promotes cell growth, migration and invasion in LUSC by targeting FGF9. However, the physiological function of dysregulation of miR-497-5p in LUSC have been rarely elucidated yet. In an analysis, miR-497-5p may participate in LUSC progression and been down-regulated in LUSC patients [9, 10].

Cell division cycle associated protein 4 (CDCA4), can function on cell proliferation and p53-dependent transcriptional activation [11]. Previous studies show that overexpression of SEI members leads to inhibition of p53-dependent growth [11]. SEI-1 and SEI-2 take part in in early E2F2-mediated cell cycle progression and tumorigenesis [12]. E2F family transcription factors participate in biological functions of cells, including cell cycle, DNA repair, development, differentiation, and metabolism [13]. However, DNA damage induces E2F-1 to bind to tumor suppressor gene p53 to stimulate apoptosis [14]. On this basis, further research on the CDCA protein family may lead to a better understanding of its function in LUSC.

This investigation investigated biological function of CDCA4/miR-497-5p in the carcinogenesis of LUSC, which might offer a rationale for LUSC therapy.

## Materials and methods

### Microarray analysis

Data of mature miRNAs and mRNAs in The Cancer Genome Atlas (TCGA)-LUSC dataset were acquired from TCGA (https://portal.gdc.cancer.gov/) on February 12, 2020. After screening, miRNA data (cancer: n = 473; normal: n = 45) and data of mRNA sequences (cancer: n = 497; normal: n = 49) were obtained. Differentially expressed mRNAs (DEmRNAs) were identified by R package “edgeR” (|logFC|> 2, padj < 0.05) [15]. After determining the miRNA, and miRTarBase (http://mirtarbase.mbc.nctu.edu.tw/php/index.php), miRDB (http://mirdb.org/), TargetScan (http://www.targetscan.org/vert_72/), starBase (http://starbase.sysu.edu.cn) /), and mirDIP (http://ophid.utoronto.ca/mirDIP/) were used for target gene prediction [16–20]. The predicted results were intersected with DEmRNAs. The correlation between miRNA and mRNA was calculated. The miRNA-mRNA regulatory pair was determined.

### Cell culture

Lung epithelial cells BEAS-2B (ATCC®CRL-9609™), LUSC cells NCI‐H520 (ATCC®HTB-182™), NCI-H1703 (ATCC®CRL-5889™), SK-MES-1 (ATCC®HTB-58™), and EPLC-32M1 (ATCC®CRL-2182™) were obtained from American Type Culture Collection (ATCC). They were grown in Roswell Park Memorial Institute (RPMI) 1640 medium with 10% fetal bovine serum (FBS) (Invitrogen) in a moist incubator.

### Cell transfection

miR-NC/MiR-497-5p mimic (miR-mimic) (RiboBio; Guangzhou, China) as well as pcDNA3.1-CDCA4/pcDNA3.1(Invitrogen; Carlsbad, CA, USA) were transfected into NCI‐H520 cells using Lipofectamine 3000 (Invitrogen).

### qRT-PCR

Total RNA was extracted using TRIzol kit (Invitrogen). For miR-497-5p detection, complementary DNA (cDNA) was synthesized using TaqMan reverse transcription kit (Applied Biosystems) and amplified using TransScript Green One-Step qRT-PCR SuperMix (Qiagen). For CDCA4 mRNA detection, total RNA was reversely transcribed into cDNA using the High-Capacity cDNA Reverse Transcription Kit (Applied Biosystems, Foster City, CA, USA). Kit used for qPCR Taq was SYBR Premix Ex (Qiagen, Valencia, CA, USA). The relative expression of miR-497-5p and CDCA4 mRNA was analyzed by $2^{{ - \Delta \Delta {\text{Ct}}}}$ method, and was standardized by U6 or GAPDH. Primers used are shown in Table 1.

**Table 1:** qRT-PCR primer sequences

Gene	Primer sequences (5’ → 3’)	
miR-497-5p	F: CCTTCAGCAGCACACTGTGG	R: CAGTGCAGGGTCCGAGGTAT
U6	F: CTCGCTTCGGCAGCACA	R: ACGCTTCACGAATTTGCGT
CDCA4	F: ATTTGAAACGCTGGAGACT	R: CCCATCATGCCTGTCAGTA
GAPDH	F: TGACTTCAACAGCGACACCCA	R: CACCCTGTTGCTGTAGCCAAA

### CCK-8 assay

In short, transfected NCI‐H520 cells were seeded to 96-well plates (3 × 10^3^ cells/well), followed by incubation routinely. Next, 20 μL CCK-8 buffer was added to each well at 0/24/48/72/96 h, respectively, followed by incubation for 2 h. Then, optical density (OD) value at 490 nm was measured on a microplate reader.

### Scratch healing assay

Tip of a 10 µL sterile pipette was applied to scrape the formed cell monolayer in plates, and phosphate buffer saline (PBS) was used to wash off the separated cells. After 0 and 24 h, wound width was observed and photographed. Wound healing rates of different treatment groups were collected and calculated.

### Transwell invasion assay

200 µL cell suspension was injected into the upper chamber, which was coated with 1 mg/mL Matrigel gel (356234, BD Corporation, USA). The lower chamber contained 600 µL RPMI-1640 with 10% FBS. After 48 h of incubation at 37 ℃, cells on the lower surface were fixed with 4% formaldehyde for 15 min and stained with 0.5% crystal violet for 30 min. Then, cells passing through the membrane were observed, counted and photographed by a microscope.

### Western blot assay

Total proteins were isolated using RIPA buffer (Beyotime Institute of Biotechnology, China). After quantified by bicinchoninic acid assay (BCA) (Beyotime Institute of Biotechnology, China), equivalent amount of proteins (30 µg) were separated by 10% sodium dodecyl sulfate–polyacrylamide gel electrophoresis (SDS-PAGE) and then transferred to a polyvinylidene fluoride membrane. The membrane was then incubated with primary antibodies at 4 ℃ overnight. Primary antibodies included rabbit anti-CDCA4 (Abcam, British) and rabbit anti-GAPDH (Abcam, British). The membrane was then incubated with horseradish peroxidase-conjugated goat anti-rabbit antibodies (Abcam, British). Thereafter, protein signals were detected using the enhanced chemiluminescence western blotting reagent (GE Healthcare Life Sciences, Canada). Finally, Quantity One software (version 4.62; Bio-Rad Laboratories, Inc. USA) was used for gel analysis.

### Dual-luciferase assay

Sequences on wild-type (WT) CDCA4 3’UTR and mutant (MUT) CDCA4 fragments were synthesized by GenePharma (Shanghai, China) and inserted into the pmirGLO luciferase vectors (Promega Corporation) to generate WT-CDCA4 and MUT-CDCA4. NCI‐H520 cells were co-transfected with WT-CDCA4/MUT-CDCA4 (1.6 µg) and miR-mimic/miR-NC (50 nM). Luciferase activity was assessed 48 h after culture using the dual-luciferase reporter assay system (Promega Corporation).

### Flow cytometry

Cells in proper density were seeded to 6-well plates and transfected in standard conditions. Cells were collected and rinsed 3 times with cold PBS, followed by resuspension in 1 × binding buffer. Afterwards, cells were treated with Annexin V-FITC and propidium iodide (PI) for 15 min at room temperature. Finally, cell apoptosis was assessed with flow cytometry (BD Biosciences, San Jose, CA, USA).

### Statistical analysis

Analysis between two groups was Student’s *t*-test. Analysis among multiple groups were One-way analysis of variance. Student’s *t*-test was used for post hoc test Pearson correlation coefficient between two genes was calculated. Statistical analysis was conducted on GraphPad Prism 6.0 (La Jolla, CA). Each assay was repeated 3 times. *P* < 0.05 denoted statistically significant.

## Results

### Low level of miR-497-5p in LUSC

**Fig. 1:**
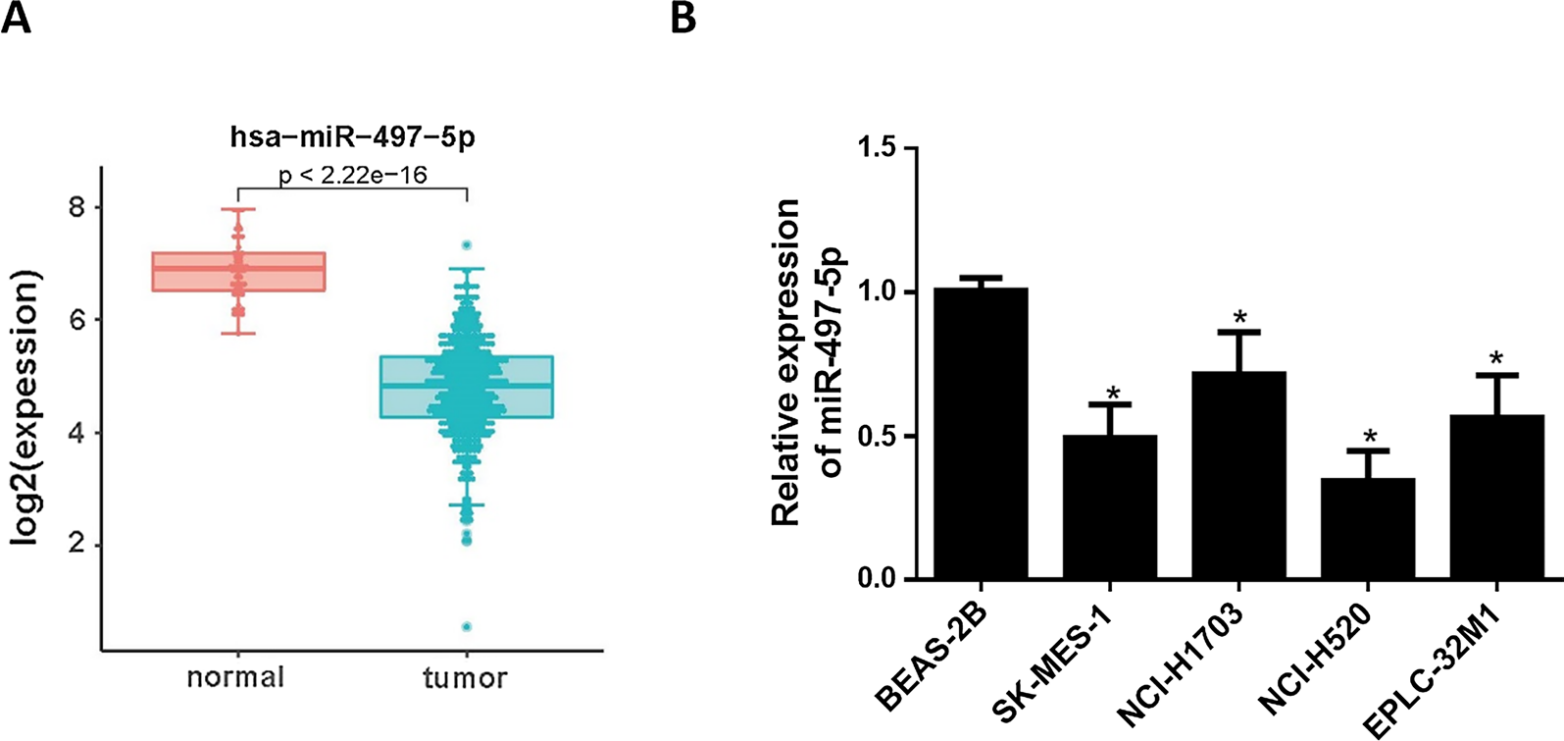
Downregulated miR-497-5p in LUSC. **A** MiR-497-5p expression in database; **B** MiR-497-5p relative expression; *indicates *p* < 0.05

Firstly, bioinformatics data suggested remarkably low expression of miR-497-5p in LUSC tissue (Fig. 1A). A study exhibited that overexpressing miR-497-5p restrains progression of non-small cell lung cancer (NSCLC) cells [21]. That is why it was studied here. qRT-PCR result indicated that miR-497-5p in LUSC cell lines was significantly downregulated (Fig. 1B). Nonetheless, this gene was markedly relevant to patient’s survival (Additional file 1: Fig. S1A). Since NCI‐H520 had the lowest level of miR-497-5p, NCI-H520 was selected for later experiments.

### Overexpressed miR-497-5p restrains LUSC progression

**Fig. 2:**
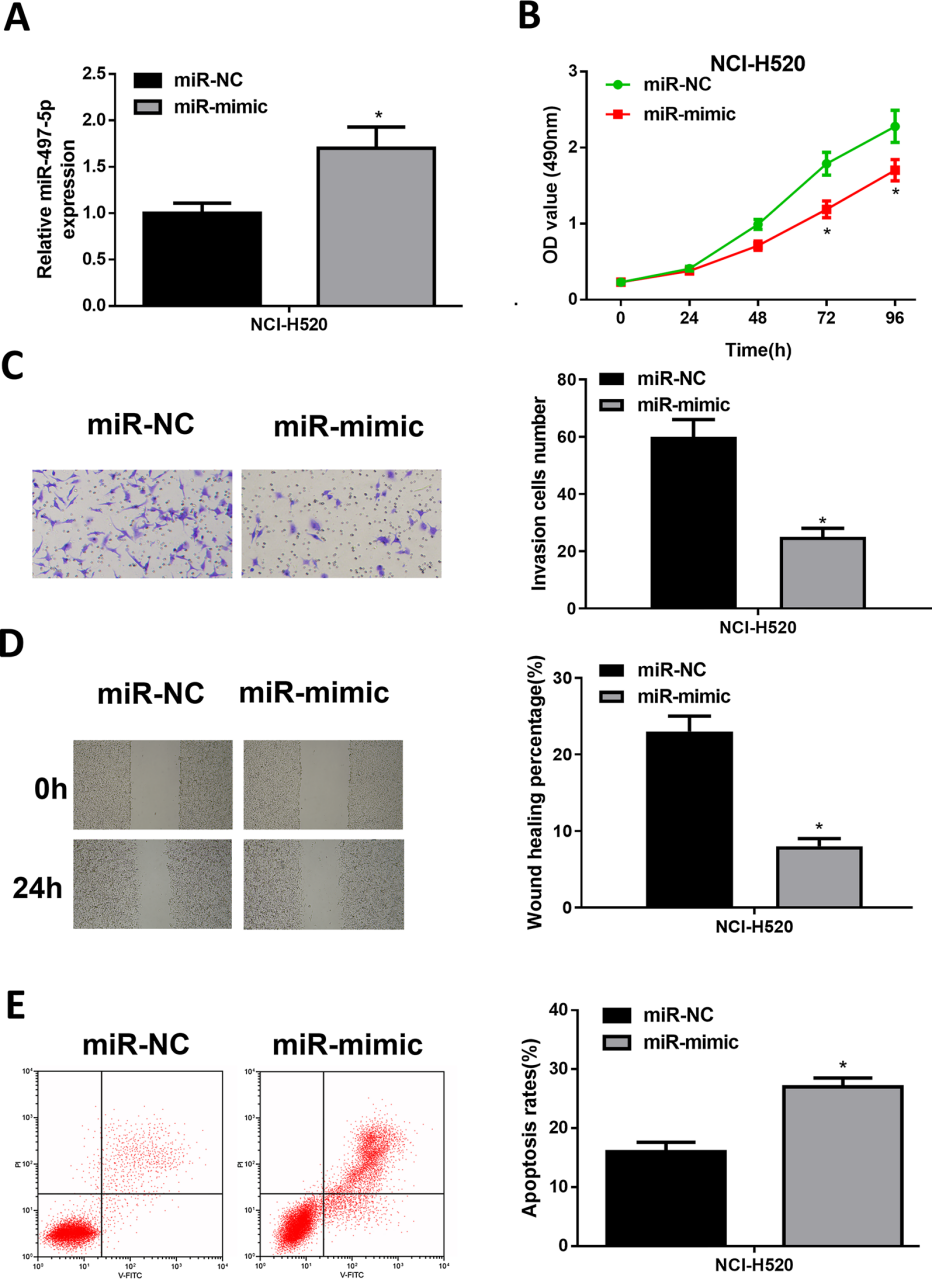
Overexpressed miR-497-5p restrains LUSC progression. **A** Expression of miR-497-5p in miR-mimic or miR-NC; **B** Viability of NCI‐H520; **C** The results Transwell assay (100 ×); **D** The results of scratch healing (40 ×); **E** The apoptotic rate of NCI‐H520; *denotes *p* < 0.05

NCI‐H520 cell line with overexpressed miR-497-5p was constructed. Based on qRT-PCR, miR-497-5p was markedly increased in the constructed cell line, suggesting it could be used for subsequent experiments (Fig. 2A). Based on CCK8, overexpressing miR-497-5p triggered a conspicuous reduction of the proliferative ability of NCI‐H520 cells compared to that of the negative control (Fig. 2B). Transwell assay and scratch healing method demonstrated that miR-mimic group displayed a conspicuously restrained invasion and migration of cancer cells (Fig. 2C–D). Flow cytometry showed a remarkable elevation of cancer cell apoptosis (Fig. 2E). Taken together, miR-497-5p overexpression repressed cancer-related behaviors.

### MiR-497-5p represses CDCA4 expression in LUSC

**Fig. 3:**
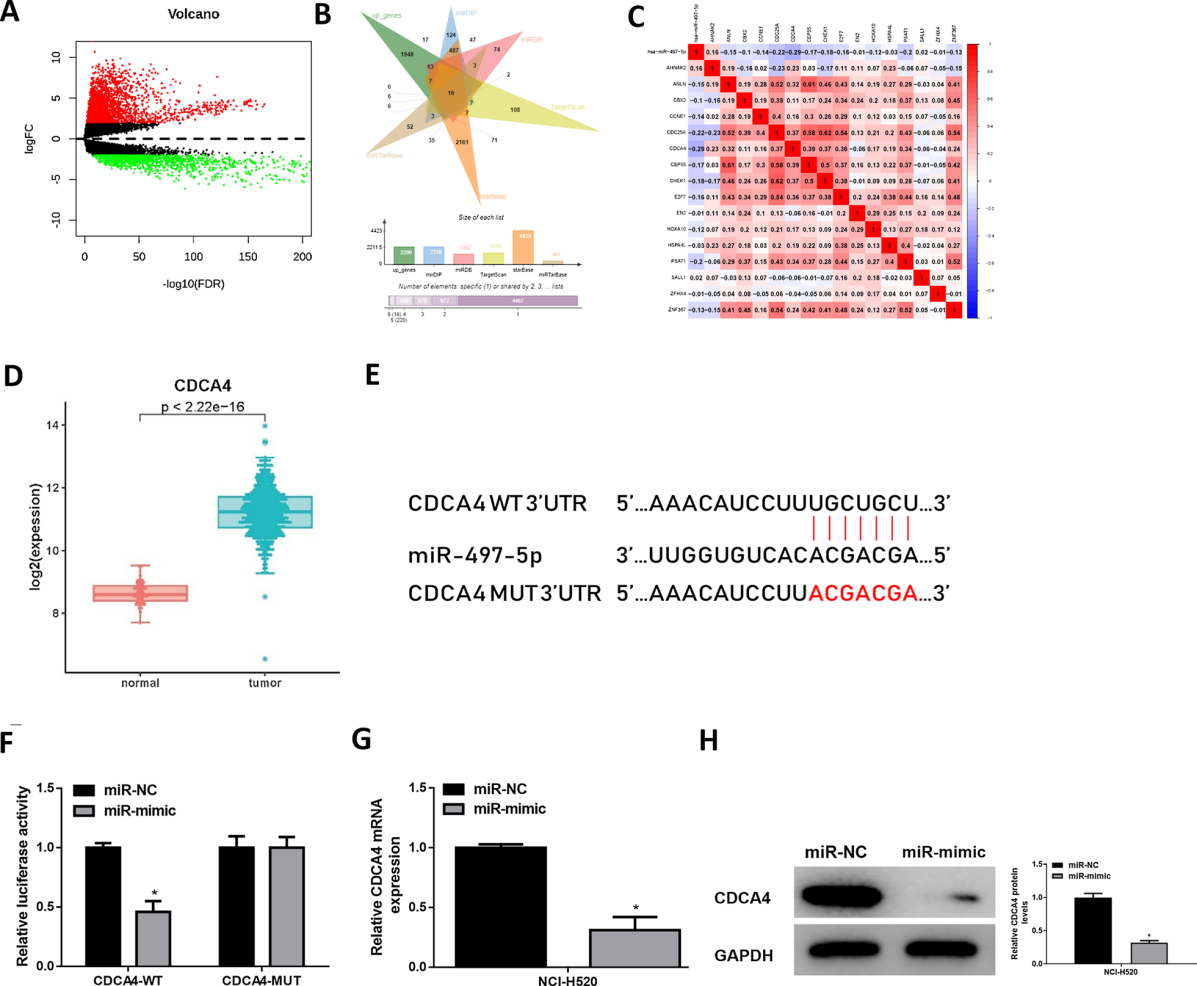
MiR-497-5p restrains CDCA4 in LUSC cells. **A** Volcano plot of DEmRNAs from TCGA-LUSC; **B** Venn diagram shows the intersection between downstream target genes and differentially up-regulated mRNAs; **C** Pearson correlation between two genes; **D** The relative level of CDCA4; **E** The binding sites between two genes; **F** Luciferase activity of NCI‐H520; **G** The expression of CDCA4 mRNA; **H** The expression of CDCA4 protein; *denotes *p* < 0.05

Downstream targets of miR-497-5p were explored. Firstly, differential analysis was performed for the merged mRNA FPKM and GTEx data, and 2,312 DEmRNAs were obtained (Fig. 3A), among which 2,290 were up-regulated. Then, targets of miR-497-5p were simultaneously predicted using databases, and an intersection of the obtained predicted target genes and the 2,290 up-regulated DEmRNAs was taken to acquire 16 DEmRNAs with binding sites of miR-497-5p (Fig. 3B). CDCA4 possessed the highest Pearson correlation coefficient with the researched miRNA and showed a significantly negative correlation (Fig. 3C). TCGA-LUSC data showed that CDCA4 was highly expressed in LUSC tissue, but survival was not remarkably impacted (Fig. 3D, Additional file 1: Fig. S1B). Moreover, CDCA4 level was not conspicuously different in different T stages and M stages of patients. CDCA4 level of N1 + N2 + N3 patients was higher than N0 patients (*p* = 0.064; not remarkably different). Stage III and Stage IV patients showed notably higher CDCA4 expression than Stage I and Stage II patients (*p* = 0.002) (Additional file 1: Fig. S1C). Next, molecular effect of miR-497-5p on CDCA4 was further verified, and their binding sites were shown in Fig. 3E. Subsequently, luciferase method revealed that miR-497-5p upregulation attenuated luciferase intensity of cells with CDCA4-WT instead of CDCA4-MUT, indicating targeting between two genes (Fig. 3F). In addition, levels of CDCA4 protein and mRNA in the miR-497-5p overexpression group were significantly decreased as tested by western blot and qRT-PCR methods (Fig. 3G–H). These data suggested that CDCA4 could serve as a downstream target of miR-497-5p in LUSC.

### MiR-497-5p restrains development of LUSC by down-regulating CDCA4

**Fig. 4:**
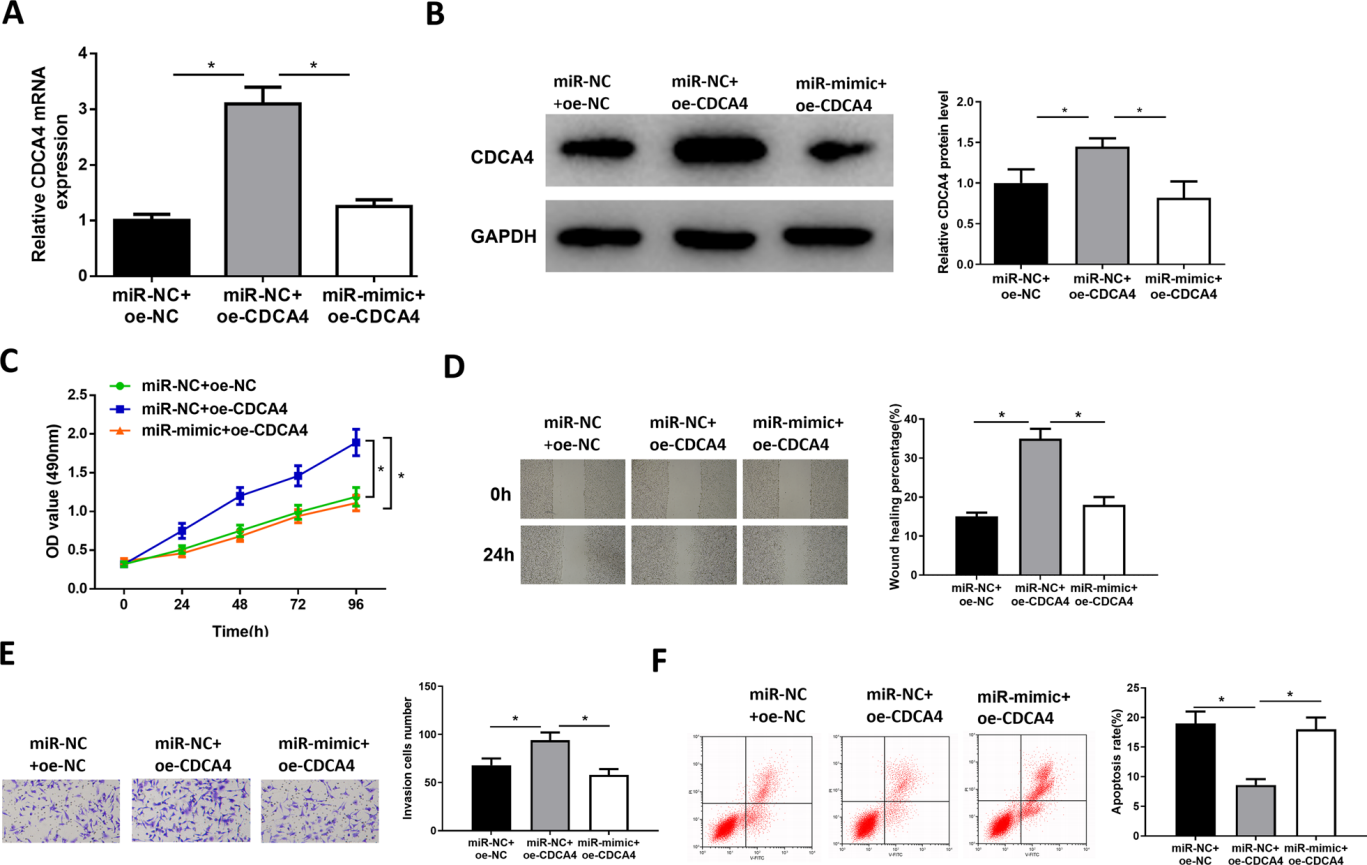
MiR-497-5p represses cancer progression by down-regulating CDCA4. **A**,**B** Levels of CDCA4 mRNA and protein; **C** The viability of NCI‐H520 cells after transfection; **D** The scratch healing of NCI‐H520 cells after transfection (40 ×); **E** The invasion of cells after transfection (100 ×); **F** The apoptotic rate of NCI‐H520; *denotes *p* < 0.05

Cells were transfected to perform rescue assay. The level of CDCA4 was increased notably in miR-NC + oe-CDCA4 group, while it was restored in miR-mimic + oe-CDCA4 (Fig. 4A–B). CCK8 assay results uncovered that the enhanced proliferative ability in LUSC induced by overexpression of CDCA4 could be counteracted by miR-mimic (Fig. 4C). Subsequently, experiments showed that overexpression of CDCA4 noticeably enhanced the invasion and migration in LUSC, while oe-CDCA4 + miR-mimic eliminated these effects (Fig. 4D–E). In addition, flow cytometry results confirmed that CDCA4 significantly inhibited cancer cell apoptosis, while overexpressing miR-497-5p reversed the inhibitory effect (Fig. 4F). In summary, miR-497-5p inhibited LUSC progression by down-regulating CDCA4.

## Discussion

Most NSCLC patients, including LUSC patients, have advanced cancer by the time they are diagnosed with cancer [22]. Therefore, the prognosis of LUSC patients is poor [23]. Studies show that different miRNAs can promote or inhibit LUSC [24]. For example, miR-448 can regulate metastasis of LUSC cells by mediating DCLK1 [7]. MiR-372‐3p accelerates growth and metastasis of LUSC via mediating FGF 9 [8]. This paper investigated the functions of miR-497-5p on LUSC cells.

MiRNAs may be applied to evaluate NSCLC [25, 26]. However, compared with other cancer species, there are still few studies on mechanism of miRNA affecting occurrence of LUSC. MiR-497-5p was less expressed in LUSC tissue according to microarray analysis. Meanwhile, some cellular experiments also proved inhibition of miR-493-5p on LUSC cells, which coincided with the earlier investigations. For instance, Guo et al. found that by modulating IGF1, miR-497-5p represses metastasis of liver cancer [27]. Upregulated miR-497-5p inhibits colorectal cancer cell growth by targeting PTPN3 [28]. Here, we demonstrated miR-497-5p a tumor suppressor factor as proved by bioinformatics analysis and cellular experiments. This result further confirmed and perfected the theory that miR-497-5p affects a variety of human cancers as an inhibitor.

We found that down-regulated miR-497-5p might target CDCA4. According to existing studies, CDCA4 can regulate cell transcriptional activation and cell proliferation [29]. For example, reducing CDCA4 expression can inhibit the proliferation of MDA-MB-231 cells in triple negative breast cancer [30]. MiR-15a-5p reduces invasiveness of melanoma cells through mediatingCDCA4 [31]. CDCA4 was uncovered to be highly expressed in LUSC cells here. Moreover, CDCA4 was a target gene of miR-497-5p via bioinformatics methods. On this basis, their binding was verified. Overexpressing CDCA4 deteriorates LUSC progression. In addition, the rescue assay result demonstrated that miR-497-5p repressed cancer-related functions by restraining CDCA4. These results indicated that CDCA4 was regulated by miR-497-5p during cancer progression. However, their expressions were not relevant to patient’s survival. This might due to that the expressions of two genes were mediated by other cells in tumor microenvironment, thus showing no notable differences in varying severe levels of patients. We will arrange further assays to investigate the possible reasons.

In sum, the results demonstrated that miR-497-5p restrained the progression of LUSC cells. In addition, in terms of mechanism, miR-497-5p targets 3’UTR region of CDCA4 mRNA and miR-497-5p plays an anti-tumor role by down-regulating CDCA4 level in LUSC cells, which casts light on LUSC pathogenesis.

## Supplementary Information

**Additional file 1: Fig. S1.** Prognostic performance of miR-497-5p and CDCA4 on patients in TCGA database. A-B: Survival curve of miR-497-5p and CDCA4 in LUSC patients. C: CDCA4 expression in LUSC patients at different stages.

## Data Availability

The data used to support the findings of this study are included within the article. The data and materials in the current study are available from the corresponding author on reasonable request.

## Abbreviations

LUSC: Lung squamous cell carcinoma
CDCA4: Cycle-associated protein 4
NSCLC: Non-small cell lung cancer
miRNAs: MicroRNAs
ATCC: American Type Culture Collection
RPMI: Roswell Park Memorial Institute
FBS: Fetal bovine serum
cDNA: Complementary DNA
snRNA: Small nuclear RNA
GAPDH: Glyceraldehyde 3-phosphate dehydrogenase
PBS: Phosphate buffer saline
BCA: Bicinchoninic acid assay
WT: Wild-type
ANOVA: One-way analysis of variance
