# Supplementary figures and images for: MiR-497-5p down-regulates CDCA4 to restrains lung squamous cell carcinoma progression
Source: J Cardiothorac Surg. 2021 Nov 12;16:330. doi: 10.1186/s13019-021-01698-2 (PMC8588708; doi:10.1186/s13019-021-01698-2)

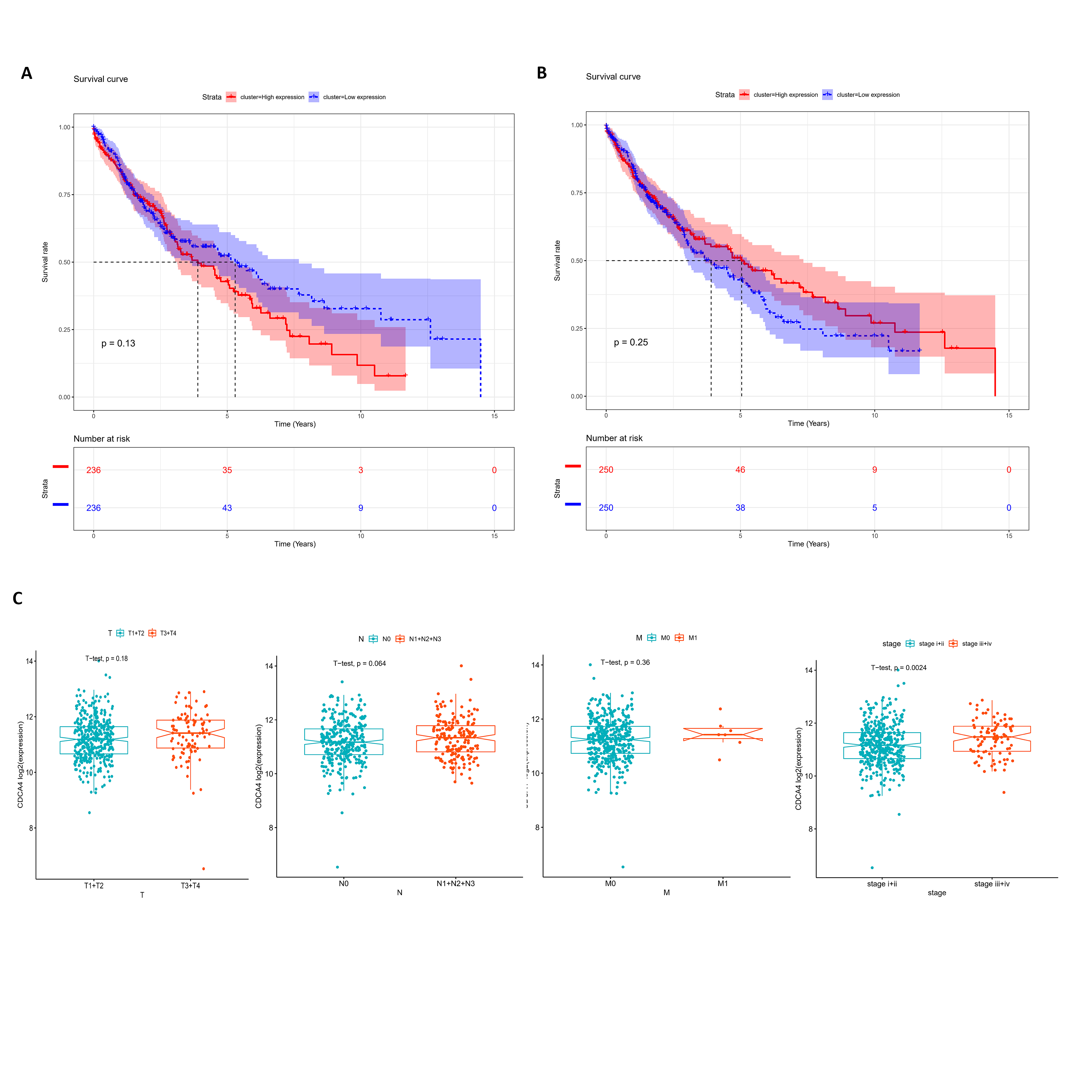

Supplement: Supplementary file 1 — Additional file 1: Fig. S1. Prognostic performance of miR-497-5p and CDCA4 on patients in TCGA database. A-B: Survival curve of miR-497-5p and CDCA4 in LUSC patients. C: CDCA4 expression in LUSC patients at different stages. [file 13019_2021_1698_MOESM1_ESM.tif]
